# Distant lymph node metastases caused by esophageal cancer invasion to the lamina propria: a case report

**DOI:** 10.1186/s40792-016-0271-1

**Published:** 2016-11-30

**Authors:** Satoshi Tsutsumi, Hiroshi Saeki, Yuichiro Nakashima, Yu Nakaji, Kensuke Kudou, Ryosuke Tsutsumi, Sho Nishimura, Shingo Akiyama, Hirotada Tajiri, Takafumi Yukaya, Kimihiro Tanaka, Ryota Nakanishi, Masahiko Sugiyama, Kippei Ohgaki, Hideto Sonoda, Minako Hirahashi, Eiji Oki, Masaru Morita, Yoshinao Oda, Yoshihiko Maehara

**Affiliations:** 1 Department of Surgery and Science, Graduate School of Medical Sciences, Kyushu University, 3-1-1 Maidashi, Higashi-ku, Fukuoka, 812-8582 Japan; 2 Department of Anatomic Pathology, Pathological Science, Graduate School of Medical Sciences, Kyushu University, Fukuoka, Japan; 3 Department of Gastroenterological Surgery, National Kyushu Cancer Center, Fukuoka, Japan

**Keywords:** Superficial esophageal cancer, Para-aortic lymph node metastasis, Distant metastasis, Endoscopic resection, Additional treatment

## Abstract

**Background:**

Pathological examination after endoscopic submucosal dissection revealed that a 62-year-old male had esophageal squamous cell carcinoma with lamina propria mucosal invasion and lymphatic permeation.

**Case presentation:**

The patient underwent subtotal esophagectomy and reconstruction as an additional therapy. At 3 years and 4 months after esophagectomy, enlargement of abdominal para-aortic lymph nodes metastases was detected by computed tomography scanning. A total of 50.4 Gy of radiation and two cycles of 5-fluorouracil plus cisplatin were administered. The lymph node metastases were markedly reduced by chemoradiotherapy; however, at 1 year and 1 month later (4 years and 5 months after esophagectomy), left adrenal gland recurrence was found. Although resection was performed, the patient died from cancer progression at 5 years and 4 months after esophagectomy.

**Conclusions:**

This case demonstrates that esophageal squamous cell carcinoma with invasion to the lamina propria and lymphatic permeation has the potential to cause distant metastases.

## Background

Although lymph node recurrence is common in esophageal cancer, it is rare in tumors restricted to the mucosal layer. In Japan, superficial esophageal squamous cell carcinoma (ESCC) is classified into four subtypes: tumor in epithelium (T1a-EP); tumor invasion to lamina propria mucosa (T1a-LPM); tumor invasion to muscularis mucosa (T1a-MM); and tumor invasion to the upper/middle/lower third of the submucosal layer (T1b-SM1/2/3) [1]. Among a case series of 75 surgically resected tumors, none of the T1a-EP and T1a-LPM cases had lymph node metastasis or lymphatic permeation, whereas T1a-MM and T1a-SM1, SM2, SM3 cases had lymph node metastasis (18, 47, 36, and 62%, respectively) and lymphatic permeation (54, 70, 54, and 75%, respectively) [2]. Other reports indicate that lymphatic permeation is rare in T1a-LPM esophageal cancer, and lymphatic and vascular permeation correlate with lymph node metastasis and recurrence [3, 4].

Based on the Guidelines for the Diagnosis and Treatment of Esophageal Cancers 2012 [1], endoscopic submucosal dissection (ESD) is an absolute indication for T1a-EP/T1a-LPM permeation without lymph node or distant metastases. ESD is a relative indication for T1a-MM/T1b-SM1 permeation without lymph node or distant metastases, depending on size and location for complete tumor extraction. For T1a-MM/T1a-SM with lymphovascular permeation, additional treatment such as surgery and chemoradiotherapy (CRT) should be considered because the presence of microscopic lymph node metastasis cannot be eliminated, even if lymph node metastasis is not detected [5].

This report describes a case of ESD followed by an additional esophagectomy for T1a-LPM ESCC with lymphatic permeation, in which distant metastasis was found over 3 years after surgery.

## Case presentation

**Fig. 1:**
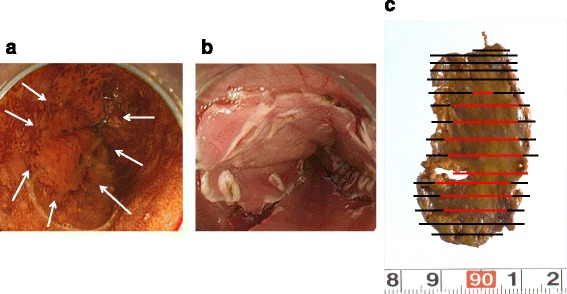
Clinical findings. **a** Endoscopic findings of 0-IIc type Lugol-voiding lesion in the lower thoracic esophagus. **b** ESD findings. **c** Resected specimen. The *black* and *red lines* indicate the cut line and the range of tumor lesion, respectively

**Fig. 2:**
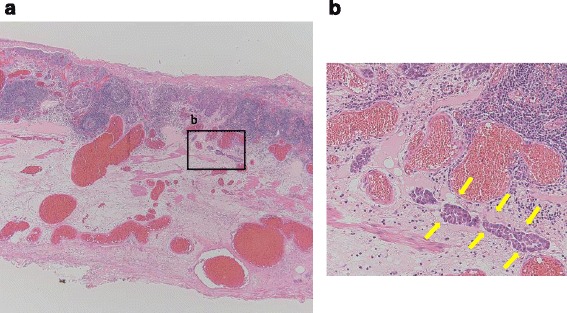
Histopathological findings. **a** Histopathological data reveal well-differentiated squamous cell carcinoma invading the lamina propria (T1a-LPM) (×20). **b** *Yellow arrows* indicate lymphatic permeation of tumor cells (×200)

A 62-year-old male underwent upper gastrointestinal endoscopy as a screening examination and was diagnosed with early ESCC. A 0-IIc type Lugol-voiding lesion was detected in the lower thoracic esophagus. There was no evidence of submucosal invasion by esophagography, narrow band imaging, or endoscopic ultrasonography. Pathological biopsy examination revealed well-differentiated ESCC. Computed tomography (CT) scanning detected neither lymph node nor distant organ metastases. As a result, ESD was performed as the initial therapy (Fig. 1). The pathological findings demonstrated well-differentiated ESCC with invasion of the lamina propria and lymphatic permeation (Fig. 2). No tumor cells were present in the proximal, distal, and radial margins. As additional therapy, subtotal esophagectomy and reconstruction were performed with a gastric tube via the posterior mediastinal route. Pathological examination of the resected specimen revealed no residual primary lesions and no metastases in any of the dissected lymph nodes. The patient had an uneventful postoperative course, with hospital discharge at 14 days after surgery.

**Fig. 3:**
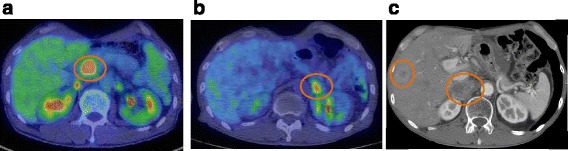
Recurrence findings. **a** Positron emission tomography-computed tomography scan findings of abdominal para-aortic lymph node recurrence at 3 years and 4 months after esophagectomy. **b** PET-CT scan of left adrenal gland recurrence at 4 years and 5 months after esophagectomy. **c** CT scan of liver and right adrenal recurrence at 5 years and 1 month after esophagectomy

At 3 years and 4 months after esophagectomy, enlargement of abdominal para-aortic lymph nodes was detected by follow-up CT scan and subsequently confirmed as recurrence of esophageal cancer by positron emission tomography (PET)-CT scanning (Fig. 3a). Two cycles of 5-fluorouracil (5-FU; 700 mg/m^2^ on days 1 to 4) combined with cisplatin (70 mg/m^2^ on day 1) and radiation (total 50.4 Gy/28 Fr) were administered, after which, the abdominal para-aortic lymph node metastases reduced markedly. At 1 year after chemoradiotherapy (4 years and 5 months after esophagectomy), left adrenal gland recurrence was found by PET-CT scan (Fig. 3b). The left adrenal gland tumor was resected. Pathological examination revealed that most of the normal adrenal cells had been replaced by well-differentiated squamous cell carcinoma cells with the same degree of differentiation as the primary lesion. At this time, the abdominal para-aortic lymph node metastases that had been treated with chemoradiotherapy were not detected by PET-CT scan; these nodes were therefore not resected. However, 5 months later (5 years and 1 month after esophagectomy), distant recurrence in the liver, right adrenal gland and abdominal lymph nodes, was recognized by CT scan (Fig. 3c). The patient died from cancer progression 3 months later (5 years and 4 months after esophagectomy).

### Discussion

Lymphatic permeation is rare in T1a-LPM ESCC. Kodama et al. reported that lymph node metastasis occurred in 3.3%, lymphatic permeation in 6.5%, and vascular permeation in 0.4% of T1a-LPM cases [3]. Yamashita et al. reported that 5-year metastasis rates for EP/LPM, MM, SM1, and SM2 ESCC were 0.4, 8.7, 7.7, and 36.2%, respectively and for mucosal cancer with and without lymphovascular permeation, 46.7 and 0.7%, respectively [4]. Because the present case was T1a-LPM ESCC with lymphatic permeation, the risk of lymph node or distant recurrence was considered to be low. Therefore, to eliminate the possibility of under-diagnosis, histopathological examination of the ESD specimen was repeated. Additional cutting of the specimen did not reveal any new malignancies, and the diagnosis was T1a-LPM ESCC with lymphatic permeation.

Because the para-aortic lymph node metastases were not resected and were therefore not examined pathologically, it was difficult to determine whether the adrenal gland metastasis was from the primary lesion or from para-aortic lymph node metastases. Of note, pathological examination of the metastatic adrenal tumor revealed that most normal adrenal cells had been replaced by well-differentiated squamous cell carcinoma cells with the same degree of differentiation as the primary lesion, suggesting that the metastatic adrenal tumor had originated from the primary lesion.

In cases of lymphatic permeation, the guidelines recommend that additional therapy such as surgical resection by esophagectomy or chemoradiotherapy should be considered. Because surgery enables the dissection of lymph nodes, it is expected to be a definite cure [6]. Motoyama et al. reported that among 17 patients with an initial clinical diagnosis of mucosal cancer without lymph node metastasis in thoracic ESCC, after ESD by additional esophagectomy with lymphadenectomy, pathological examination revealed that 5 (29%) patients had submucosal tumor invasion and the involvement of one to two lymph nodes in the lower mediastinum and abdomen. Therefore, surgical lymph node dissection is necessary and an effective treatment for this patient population [7]. However, surgery is invasive and is associated with serious risks such as postoperative complications and mortality. Esophagectomy may be challenging to perform in elderly patients, and those with cardiac or pulmonary complications. Saeki et al. reported that postoperative complications were observed in 10 of 34 patients who underwent additional esophagectomy after ESD. One patient died from pulmonary complications, but no postoperative recurrences were detected during the follow-up period [5]. Motoyama et al. reported that 4 of 17 patients (24%) who underwent esophagectomy developed an anastomotic fistula, but these were healed with conservative therapy, and surgery was not necessary. Pneumonia occurred in 3 patients (14%). Recurrent nerve palsy occurred in 4 patients (24%), but it was resolved [7]. These findings suggest that esophagectomy after ESD provides favorable disease control for ESCC with pT1a-MM/pSM and/or vessel permeation. Nevertheless, careful consideration should be given to the indication for an invasive surgical procedure.

Chemoradiotherapy is useful for patients with complications such as local and distant metastasis, and it plays a role in ESCC treatment by eliminating some micro-metastases. Yamamoto et al. reported that the overall survival rate of patients with clinical stage I ESCC was similar for chemoradiotherapy versus esophagectomy, despite a higher local recurrence rate after esophagectomy. Local carcinoma recurrences were treated by endoscopy in most patients, with no effect on overall survival [8]. Thus, chemoradiotherapy appears to be a viable alternative to esophagectomy in patients with clinical stage I ESCC. Kawaguchi et al. reported that chemoradiotherapy after ESD was an effective and safe approach for T1a-MM or T1b ESCCs. The combination of ESD and chemoradiotherapy improved the local control rate relative to chemoradiotherapy alone [9].

There is accumulating evidence on the efficacy of chemoradiotherapy for early-stage ESCC. A clinical trial conducted by the Japan Clinical Oncology Group (JCOG9702) showed that the complete response rate after chemoradiotherapy with 5-FU plus cisplatin in patients with stage I ESCC was 87.5%, and the 4-year survival rate was 80.5% [10]. These results are similar to those reported for esophagectomy in stage I patients [11]. JCOG has initiated a multicenter phase III trial (JCOG0502) to evaluate the efficacy and safety of chemoradiotherapy versus esophagectomy for clinical stage I esophageal cancer. In addition, results from a phase III trial (JCOG0508) on the efficacy of ESD combined with chemoradiotherapy for clinical stage I esophageal carcinoma are anticipated.

No definitive conclusions can be drawn from the current case concerning whether esophagectomy or chemoradiotherapy constitutes optimal treatment after ESD in patients with pT1a and vessel invasion. However, because this disease is extremely rare, it would be impractical to attempt to determine the superiority of additional treatment by conducting randomized controlled trials. Further findings from multiple institutes must be reported to enable discussion of the optimal treatment strategy.

## Conclusions

ESCC with lamina propria invasion and lymphatic permeation may have the malignancy potential to cause distant metastasis. For cases with lymphovascular permeation, even if the tumor depth corresponds to T1a-MM or LPM, it is important to be aware of the possibility of distant metastasis after additional esophagectomy. Thus, strict follow-up is required in this patient population.

## Abbreviations

CRT: Chemoradiotherapy
CT: Computed tomography
ESCC: Esophageal squamous cell carcinoma
ESD: Endoscopic submucosal dissection
JCOG: Japan Clinical Oncology Group
PET-CT: Positron emission tomography-computed tomography
T1a-EP: Tumor in epithelium
T1a-LPM: Tumor invades lamina propria mucosa
T1a-MM: Tumor invades muscularis mucosa
T1b-SM1/2/3: Tumor invades the upper/middle/lower third of the submucosal layer
